# p300 is upregulated by docetaxel and is a target in chemoresistant prostate cancer

**DOI:** 10.1530/ERC-19-0488

**Published:** 2020-01-17

**Authors:** Martina Gruber, Lavinia Ferrone, Martin Puhr, Frédéric R Santer, Tobias Furlan, Iris E Eder, Natalie Sampson, Georg Schäfer, Florian Handle, Zoran Culig

**Affiliations:** 1 Division of Experimental Urology, Department of Urology, Medical University of Innsbruck, Innsbruck, Austria; 2 Department of Biomedical, Experimental and Clinical Sciences, University of Florence, Florence, Italy; 3 Department of Pathology, Neuropathology, and Molecular Pathology, Medical University of Innsbruck, Innsbruck, Austria; 4 Molecular Endocrinology Laboratory, Department of Cellular and Molecular Medicine, KU Leuven, Leuven, Belgium

**Keywords:** prostate cancer, chemotherapy resistance, p300, docetaxel, colony formation ability

## Abstract

Administration of the microtubule inhibitor docetaxel is a common treatment for metastatic castration-resistant prostate cancer (mCRPC) and results in prolonged patient overall survival. Usually, after a short period of time chemotherapy resistance emerges and there is urgent need to find new therapeutic targets to overcome therapy resistance. The lysine-acetyltransferase p300 has been correlated to prostate cancer (PCa) progression. Here, we aimed to clarify a possible function of p300 in chemotherapy resistance and verify p300 as a target in chemoresistant PCa. Immunohistochemistry staining of tissue samples revealed significantly higher p300 protein expression in patients who received docetaxel as a neoadjuvant therapy compared to control patients. Elevated p300 expression was confirmed by analysis of publicly available patient data, where significantly higher p300 mRNA expression was found in tissue of mCRPC tumors of docetaxel-treated patients. Consistently, docetaxel-resistant PCa cells showed increased p300 protein expression compared to docetaxel-sensitive counterparts. Docetaxel treatment of PCa cells for 72 h resulted in elevated p300 expression. shRNA-mediated p300 knockdown did not alter colony formation efficiency in docetaxel-sensitive cells, but significantly reduced clonogenic potential of docetaxel-resistant cells. Downregulation of p300 in docetaxel-resistant cells also impaired cell migration and invasion. Taken together, we showed that p300 is upregulated by docetaxel, and our findings suggest that p300 is a possible co-target in treatment of chemoresistant PCa.

## Introduction

Although therapy of organ-confined prostate cancer (PCa) by radical prostatectomy or radiotherapy is curative, treatment of advanced PCa is considered merely palliative. Androgen deprivation therapy (ADT) remains the gold standard for patients with prostate-specific antigen (PSA) progression. However, tumor progression is inevitable and leads to the development of castration-resistant prostate cancer (CRPC). Treatment options for non-metastatic and metastatic CRPC (mCRPC) include inhibitors of androgen synthesis and antiandrogens such as enzalutamide and apalutamide as well as the chemotherapeutic compound docetaxel (Taxotere®).

In 2004, the landmark study TAX327 showed a significant survival benefit of 2.4 months for docetaxel treatment compared to mitoxantrone, which was the first study to demonstrate a survival benefit for chemotherapy in CRPC patients (Tannock *et al.* 2004). Docetaxel treatment resulted in PSA decline, prolonged overall survival (OS), and improved quality of life. Furthermore, the STAMPEDE and CHAARTED trials have utilized docetaxel together with ADT into first-line treatment for hormone sensitive PCa (HSPC) with a survival benefit of 13.4 months compared to ADT alone (James *et al.* 2016, Kyriakopoulos *et al.* 2018). Additionally, docetaxel treatment has no negative consequences for subsequent endocrine therapies. Both abiraterone acetate and enzalutamide are used as effective second-line therapies after resistance to docetaxel has evolved (Lavaud *et al.* 2018). Despite development of novel therapies, treatment options for mCRPC patients are still limited, and there is an urgent need to find new therapeutic targets to overcome chemotherapy resistance.

Transcriptional co-regulators of the AR are involved in therapy resistance with several of them increasingly expressed during ADT (Comuzzi *et al.* 2004, Heemers *et al.* 2007, Qin *et al.* 2014). Two of these well-known coactivators are the histone acetyltransferases p300 and CBP (CREB binding protein) that show elevated expression in advanced PCa and have oncogenic potential (Debes *et al.* 2003, Comuzzi *et al.* 2004). While these coactivators show high levels of homology, they play distinctive roles in PCa and other diseases (Kalkhoven 2004, Ramos *et al.* 2010). Common features of p300 and CBP include regulation of transcription via remodeling chromatin structure by acetylating conserved lysine amino acids of histone proteins. They are also capable of recruiting the basal transcription machinery to gene promoters and acting as adaptor molecules (Chan & La Thangue 2001). In a previous study, it was demonstrated that p300 might be a valid target in PCa cells, as downregulation of p300 induced apoptosis and decreased cell migration in androgen-dependent and CRPC cells (Santer *et al.* 2011). Based on those previous results, this study aimed to investigate whether p300 is a possible new target in the context of chemotherapy resistance. Therefore, we analyzed (1) p300 expression in patients that received docetaxel, (2) p300 expression in docetaxel-resistant (DR) cells compared to docetaxel-sensitive counterparts, and (3) the effects of short-term docetaxel treatment on p300 expression. To study the functional role of p300, an RNA-interference (RNAi) approach was used and doxycycline-inducible p300 knockdown cell lines were generated. In addition, effects of p300 downregulation on colony formation efficiency, cell migration, and invasion in docetaxel-resistant cells were determined.

## Materials and methods

### Cell culture and chemicals

Human PCa cell lines PC3 and DU145 were purchased from the American Type Culture Collection (ATCC, LGC Standards, Wesel, Germany). Docetaxel-resistant PC3-DR and DU145-DR were previously established by Puhr *et al.* (2012). CWR22RV1 and CWR22RV1-DR cells were a kind gift of Prof Dr William Watson (University College Dublin). All cell lines were cultured in RPMI 1640 (PAN Biotech, Aidenbach, Germany) supplemented with 10% (v/v) fetal bovine serum (PAN Biotech, Aidenbach, Germany), 1% (v/v) penicillin/streptomycin, and 1% (v/v) GlutaMAX (both from Lonza, Vienna, Austria). Docetaxel-resistant cell lines were cultured in the presence of 12.5 nmol/L docetaxel (Sigma Aldrich). HEK293FT cells were obtained from Life Technologies and grown according to the manufacturer’s instructions. The authenticity of all cell lines was validated via short tandem repeat (STR) profiling.

### Immunohistochemistry (IHC)

For IHC staining, a tissue microarray (TMA) of 14 patients that received neoadjuvant docetaxel therapy before radical prostatectomy and 14 patients with no chemotherapy was used. The use of patient material was approved by the Ethics Committee of the Medical University of Innsbruck (study No AM 3174 including amendment 2). For detailed information about clinical data from patients, see publication of Puhr *et al.* (2012). IHC staining was performed on a Discovery-XT staining device (Ventana) and the following specific antibody was used: anti-p300 (1:100, D8Z4E, Cell Signaling Technology). Antibody specificity was verified by Western blot and IHC staining of PC3-DR cells with p300 downregulation. For IHC, cells were embedded by coagulation in plasma clots after harvesting, transferred into a biopsy histosette, fixed in formalin, and embedded in paraffin. Importantly, cross staining of CBP was excluded by Western blot analysis.

### Transcriptome analysis of patient data

The publicly available transcriptome dataset GSE77930 (Kumar *et al.* 2016) was downloaded from the GEO database and analyzed with the Qlucore Omics Explorer v3.5. Gene set activity scores for the Hallmark ‘Androgen response’ and ‘Myc targets’ gene sets (Molecular Signatures Database, MSigDB) were calculated in R with the GSVA package (Hanzelmann *et al.* 2013).

### Western blot

Cells were lysed in LDS sample buffer and 50 µg total protein was separated either on 3–8% Tris-Acetate gels (Thermo Fisher Scientific) for analysis of p300 and CBP expression or 4–12% Bis-Tris gels (Expedeon, San Diego, CA, USA) for all other proteins and transferred onto methanol-activated PVDF membranes or 0.2 µm nitrocellulose membranes (GE Healthcare). Blocking of membranes and antibody incubation were performed in 5% BSA in TBS-T. The following antibodies were used: anti-p300 (1:4000, ab10485, Abcam), anti-CBP (1:1000, Cell Signaling Technology), anti-c-Myc (1:1000, Cell Signaling Technology), anti-Histone H3 (1:1000, Cell Signaling Technology), anti-Acetyl-Histone H3 (Lys18, 1:1000, Cell Signaling Technology), anti-Vimentin (1:500, Santa Cruz Biotechnology), anti-Vinculin (1:500, Santa Cruz Biotechnology), anti-Lamin A (1:2000, Abcam), anti-α-tubulin (1:500, Santa Cruz Biotechnology), and anti-GAPDH (1:50000, Merck Millipore). House-keeping controls were selected in a cell line-specific manner on the basis of data showing no change in their expression in that cell line.

### RNA isolation and quantitative real-time PCR

Total RNA was isolated using the EXTRACTME TOTAL RNA KIT (LabConsulting, Vienna, Austria) according to the manufacturer’s manual. cDNA synthesis was performed with the iScript Select cDNA Synthesis Kit (Bio-Rad). For real-time PCR a Luna Script RT Super Mix Kit (New England Biolabs, Ipswich, MA, USA) was used. HPRT1 (Fwd: GCTTTCCTTGGTCAGGCAGTA, Rev: GTCTGGCTTATATCCAACACTTCGT, Probe: CAAGGTCGCAAGCTTGCTGGTGAAAAGGA), TATA-Box binding protein (TBP; Fwd: CACGAACCACGGCACTGATT, Rev: TTTTCTTGCTGCCAGTCTGGAC, Probe: TCTTCACTCTTGGCTCCTGTGCACA), and HMBS (TaqMan Gene Expression Assay from Thermo Fisher Scientific; Hs00609297_m1) were used as reference genes. The following Taqman gene expression assays were used: p300 (Hs00914223_m1), CBP (Hs00932878_m1), c-Myc (Hs00153408_m1), and Vimentin (Hs00185584_m1).

### Generation of doxycycline-inducible knockdown cell lines

Stable cell lines with inducible p300 knockdown were generated by lentiviral-based transduction of shRNA vectors using the BLOCK-iT HiPerform Lentiviral Pol-II miR RNAi Expression System with emGFP from Invitrogen. Briefly, miR Select oligos (Hmi405238: shp300-1; Hmi405239: shp300-2) were purchased from Life Technologies and ligated into pcDNA 6.2-GW/EmGFP-miR expression vector according to the manufacturer’s protocol. Then the shRNAs were shuttled into the pDONR221 vector to generate entry clones. Entry plasmids together with a pENTR-tetOn (from pHR-TetCMV-eGFP-dest ligated into pENTR 5′/CMVp vector) were then used in MultiSite Gateway reactions with pLenti6.4/R4R2/V5-DEST to generate doxycycline-inducible shp300-1 and shp300-2. To generate cell lines stably expressing the doxycycline activator/repressor cassette, HEK293FT cells were first co-transfected with the packaging vectors pVSV-G and psPAX2 together with pHR-SFFV-rtTAM2-T2A-Puro using X-tremeGENE reagent (Roche) according to the manufacturer’s manual. Supernatants containing virus particles were collected 48 h after transfection, filtered through a 0.45 µm membrane filter, and used for infection of target cells. Cells were selected with 2 µg/mL puromycin and subsequently transfected with packaging vectors together with the pLenti6.4 expression vector as described previously. Selection of infected cells was performed using 2 µg/mL blasticidine. For activation of the system, 100 ng/mL doxycycline was used. Activation status of the system was verified by GFP expression.

### Proliferation assay

Cumulative population doubling levels (PDL) were determined by continuously seeding a defined number of cells in T25 flasks. Cell numbers were determined by CASY cell counter (Schärfe System, Reutlingen, Germany) every 3–4 days. Cumulative PDL was calculated with the following formula: PDL = 3.32 × (log10(X) − log10(Y)) + I, where X = number of cells at the end of growth period, Y = number of cells at the beginning of growth period, and I = initial population doubling level. To compare the growth curves, linear fit regression with the test for significance of slopes and intercepts was performed in GraphPad Prism 8.

### High-resolution colony formation assay

Cell numbers were determined using CASY cell counter system (Schärfe System). Per 75 cm^2^ culture flask, 1000 cells were seeded and incubated for 10–14 days in the absence or presence of 100 ng/mL doxycycline. Cells were fixed with 100% ice-cold methanol for 5 min and stained with crystal violet (0.5% dissolved in PBS containing 20% methanol (Sigma)) for 5 min. Colony formation efficiency was determined by the software CATCH-colonies (https://catch-colonies.net). To correct for differences in the seeding density of the different stable shRNA cell lines, the colony formation efficiency was normalized to flasks that were seeded at the same time but not treated with doxycycline.

### Wound scratch assay

Cells were seeded until they were nearly confluent in multi-well plates and treated with 100 ng/mL doxycycline or 10 µM CPI-637 (MedChem Express, Monmouth Junction, NJ, USA) for 96 h in total. After the first 24 h, 10 µM of the proliferation inhibitor cytosine β-D-arabinofuranoside was added. After another 24 h, a scratch was made using a 10-µL pipette tip. Images were taken after another 48 h and analyzed using the MRI Wound Healing Tool of ImageJ.

### Migration and invasion assay

For migration and invasion assays, Boyden chamber inserts with 8 µm pore size (Fluoroblok System, Becton Dickinson) were used. 3 × 10^4^ cells per well of PC3-DR shCtrl, PC3-DR shp300-1, and PC3-DR shp300-2 were seeded in duplicate in serum-free medium. Bottom chambers were filled with medium containing 10% FCS serving as chemoattractant. For invasion assays, inserts were coated with 30 µl of matrigel (diluted 1:3 in serum-free medium; Corning). Cells were incubated for 48–72 h in the absence or presence of 100 ng/mL doxycycline or 20 µM CPI-637, respectively. Afterwards, cells were stained with 2 µM of calcein AM (Sigma) diluted in HBSS containing 1% FCS. Fluorescent images were taken using a JuLI smart fluorescent cell analyzer (Science Services, Munich, Germany), and extinction/emission at 494/517 was measured using TECAN plate reader (Tecan Group Ltd., Männedorf, Switzerland).

### Immunofluorescence

Cells were seeded on glass coverslips in the absence or presence of 100 ng/mL doxycycline for 72 h. Cells were fixed with 4% paraformaldehyde and permeabilized with 1% BSA in PBS containing 0.2% Triton X-100. Washing steps were performed with 1% BSA in PBS. Anti-Vimentin (1:500, Santa Cruz Biotechnology) was used as a primary antibody, together with the fluorescence-labeled secondary antibody goat anti-mouse 555 (ThermoFisher). Coverslips were mounted with Vectashield mounting medium containing DAPI.

### Viability assays

PC3-DR cells were seeded in multi-well plates and treated with a range of CPI-637 concentrations. The solvent DMSO served as a control. The viability was measured using RealTime-Glo™ MT Cell Viability Assay (Promega) after 72 h and the IC50 was calculated using a nonlinear fit model with variable slope of log transformed data in GraphPad Prism 8.

### Statistical analysis

Statistical analysis was performed using GraphPad Prism 8 (GraphPad Software Inc.). Gaussian distribution of patient samples was determined using Kolmogorov–Smirnov test. Comparison of the two groups was performed using Student’s *t*-test and Mann–Whitney *U* test, depending on Gaussian distribution. Comparison of multiple groups was performed using one-way ANOVA and correcting for multiple testing using Bonferroni multiple comparison test. For integration of multiple independent replicates from methods that yield relative abundance values (qPCR, Western blot), each replicate was normalized to its average signal intensity. *P*-values of <0.05 were considered statistically significant and encoded in figure legends as follows: *P < 0.05; **P < 0.01; ***P < 0.001; and ****P < 0.0001. If not stated otherwise, doxycycline-treated cells were normalized to non-treated control cells, and doxycycline-treated cells are shown in graphs.

## Results

### p300 expression is increased in docetaxel-treated patients

**Figure 1:**
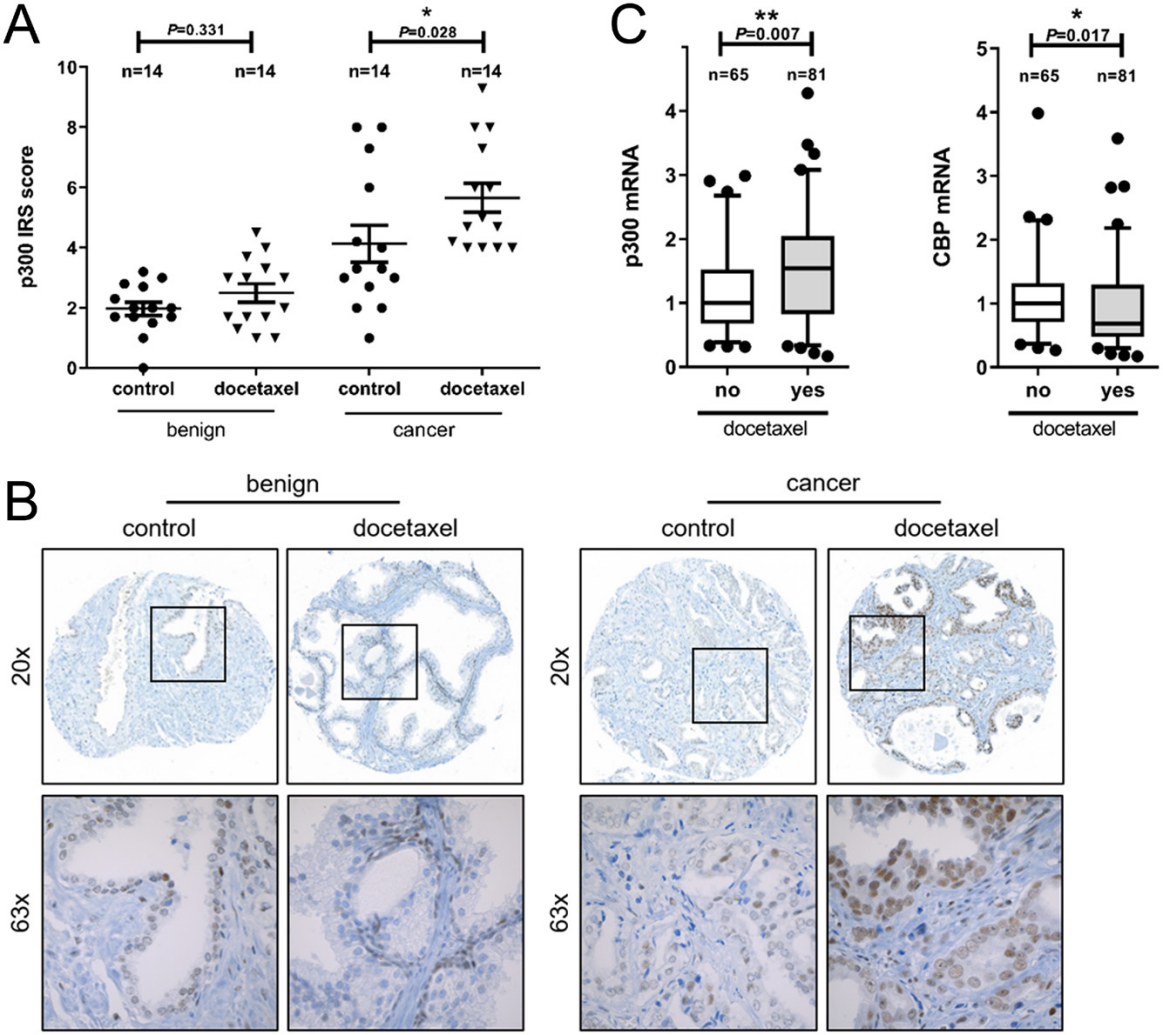
Expression of p300 is increased upon docetaxel treatment. (A) Quantification of p300 immunoreactivity scores (IRS) after IHC staining (Mann–Whitney *U* test; scatter dot blot with line at mean + s.e.m.). (B) IHC staining of p300 (nuclear localization). Upregulation of p300 expression in cancerous tissue of docetaxel-treated patients compared to control patients. (C) p300 and CBP mRNA expression were determined in samples of docetaxel-treated and docetaxel-untreated mCRPC patients (Mann–Whitney *U* test; box whisker plot with 5–95 percentile).

To evaluate whether p300 expression is altered upon docetaxel treatment, we analyzed tissue material of patients who received docetaxel by IHC staining using a specific p300 antibody (Supplementary Fig. 1A, B and C, see section on supplementary materials given at the end of this article). In total, 28 patients were included, 14 of them received neoadjuvant docetaxel therapy before radical prostatectomy (RPE), while the other 14 patients did not receive chemotherapy prior to RPE. For detailed information about selected patients, please see the work of Puhr *et al.* (2012), where clinical data are fully described. We observed a significantly increased p300 expression in cancerous areas of docetaxel-treated patients compared to control patients (Fig. 1A and B), which was not the case in benign areas. Furthermore, p300 staining was observed mainly in nuclear areas.

Elevated p300 expression was further confirmed in publicly available datasets of mCRPC tissue samples from patients that suffered from relapse after docetaxel treatment (Kumar *et al.* 2016). Our analysis revealed significantly increased p300 levels (1.5-fold) in patients treated with docetaxel compared to patients that did not receive docetaxel at any time in their treatment course (Fig. 1C). To assess if docetaxel-mediated increase is specific for p300, we included the analysis of the closely related coactivator CBP. Interestingly, CBP expression levels were found slightly decreased in docetaxel-treated patients compared to non-docetaxel-treated patients (Fig. 1C). Furthermore, we analyzed AR expression and activity upon docetaxel treatment to evaluate whether the AR has any impact on docetaxel-mediated upregulation of p300. Of note, AR mRNA expression and androgen response were not significantly changed in patients that relapsed after docetaxel treatment compared to control patients (Supplementary Fig. 2A and B).

### Docetaxel-resistant and docetaxel-treated PCa cells show increased p300 expression

**Figure 2:**
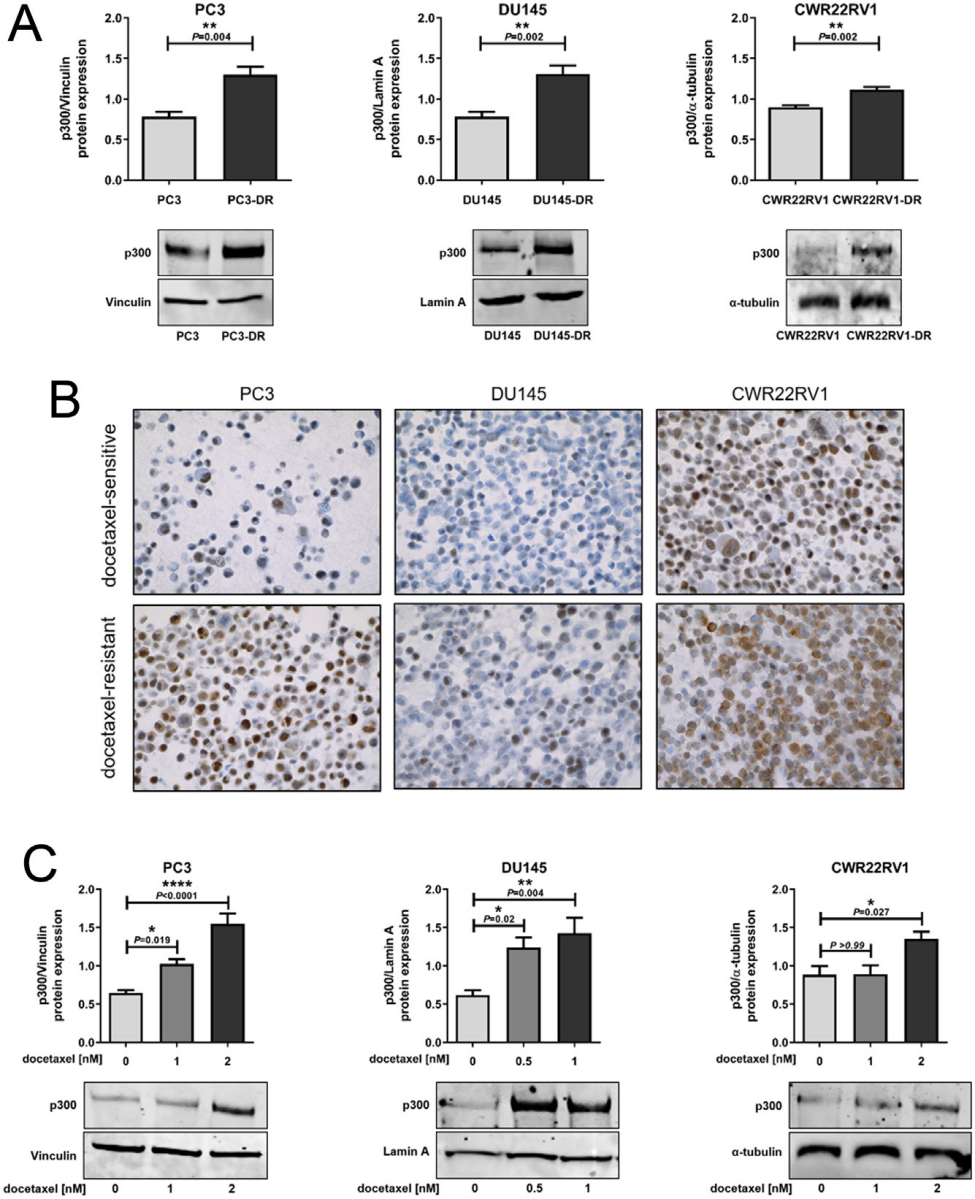
p300 expression is increased in docetaxel-resistant prostate cancer cells. (A) Comparison of p300 protein expression between docetaxel-sensitive and docetaxel-resistant (DR) PC3 (*n* = 4), DU145 (*n* = 5), and CWR22RV1 (*n* = 4). Data represent mean + s.e.m. (*t-*test). (B) IHC staining for p300 in docetaxel-resistant PC3-DR, DU145-DR, and CWR22RV1-DR compared to docetaxel-sensitive counterparts. Magnification 40×. (C) PC3 (*n* = 5), DU145 (*n* = 5), and CWR22RV1 (*n* = 4) were treated with the indicated concentrations of docetaxel for 72 h, and p300 protein expression was analyzed by Western blot, and one representative Western blot is shown. Values represent mean + s.e.m. (one-way ANOVA).

Next, we compared p300 expression in docetaxel-resistant (DR) derivatives of commonly employed PCa cell lines (PC3-DR, DU145-DR, and CWR22RV1-DR) relative to their docetaxel-sensitive counterparts. p300 protein levels were significantly increased (1.5-fold) in all three docetaxel-resistant cell lines tested compared to docetaxel-sensitive counterparts (Fig. 2A), whereas mRNA levels were unchanged (Supplementary Fig. 3A). Increased p300 protein expression in docetaxel-resistant PC3-DR and DU145-DR was confirmed by IHC staining (Fig. 2B). CBP mRNA and protein expression were not significantly regulated in docetaxel-resistant cells compared to sensitive counterparts (except downregulated CBP mRNA expression in PC3-DR cells, Supplementary Fig. 3B and C).

To understand the molecular basis of these findings, we analyzed p300 expression upon short-term docetaxel treatment in several PCa cell lines. To this end, we measured p300 mRNA and protein expression in PC3, DU145, and CWR22RV1 cells after treatment with docetaxel for 72 h. Protein expression of p300 significantly increased (2–2.5-fold increase) in all three cell lines upon docetaxel treatment (Fig. 2C), whereas mRNA levels were not significantly changed (Supplementary Fig. 4A). Time-course experiments revealed that p300 protein, but not mRNA expression, increased within 8 h of docetaxel treatment and reached a plateau after 16 h (Supplementary Fig. 4B and C). Interestingly, p300 expression in docetaxel-resistant cells decreased if the cells were cultured without docetaxel (Supplementary Fig. 4D and E). To investigate whether docetaxel influences the protein degradation rate of p300, translation was blocked with cycloheximide (CHX). Of note, p300 had a protein half-life time of around 6 h in untreated PC3 cells, whereas addition of docetaxel (PC3 and PC3-DR) stabilized the protein (Supplementary Fig. 4F).

We also analyzed the expression levels of the well-described p300 downstream target c-Myc, which is a known oncogene in PCa (Koh *et al.* 2010). Our analysis revealed no significant change of c-Myc mRNA levels (Supplementary Fig. 5A), but a Myc gene expression activity score was significantly increased in docetaxel-treated patients (Supplementary Fig. 5B). Additionally, docetaxel-resistant PC3-DR and DU145-DR showed higher c-Myc protein expression compared to docetaxel-sensitive counterparts (Supplementary Fig. 5C), and c-Myc protein expression was increased (1.5-fold) in DU145 upon treatment with 1 nM docetaxel compared to the control (Supplementary Fig. 5D).

### Docetaxel-resistant cells show reduced colony formation ability upon p300 inhibition

**Figure 3:**
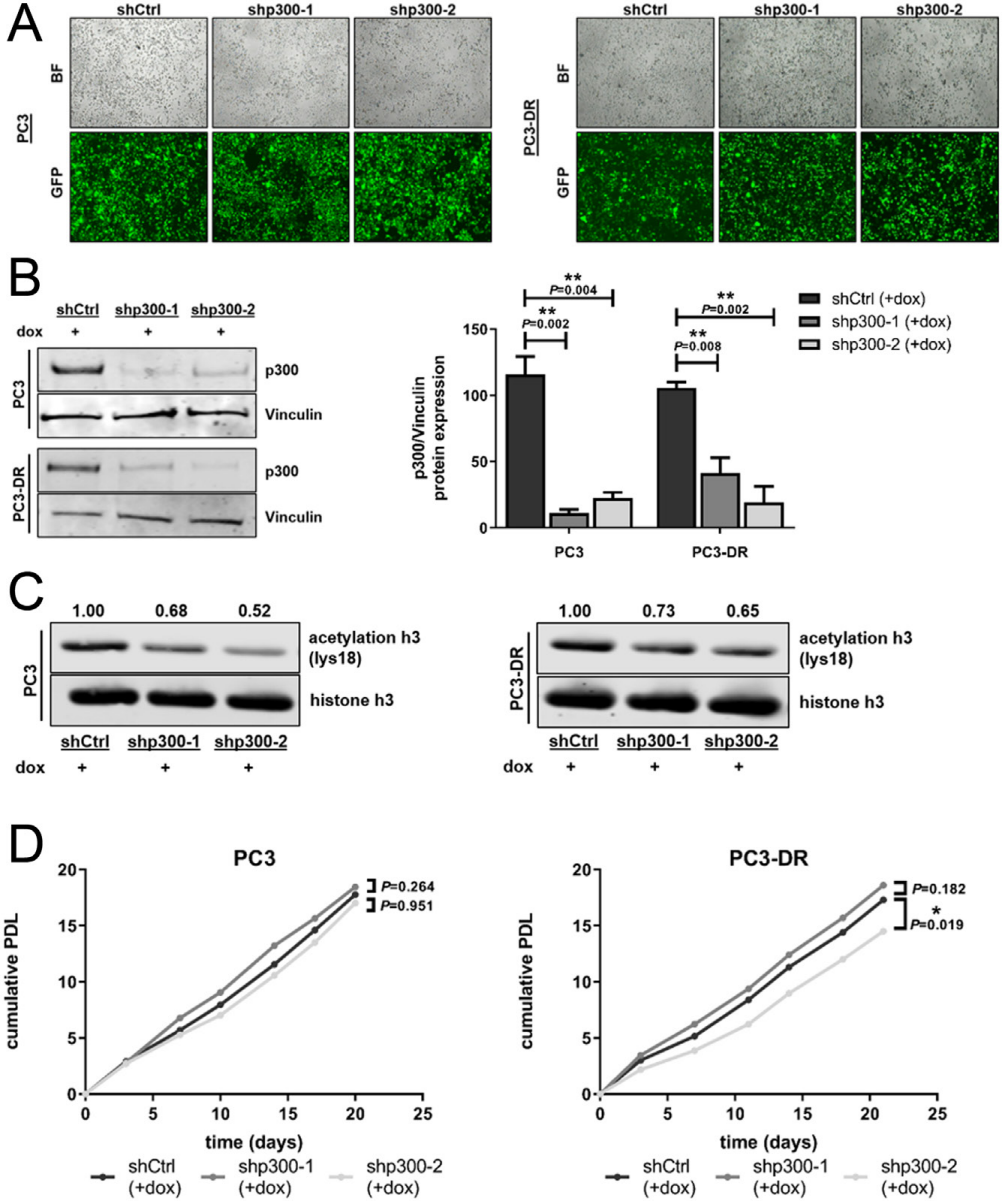
Establishment and validation of doxycycline-inducible p300 knockdown cell lines using a non-targeting control (shCtrl) sequence and two specific p300-shRNA sequences (shp300-1 and shp300-2). Docetaxel-sensitive PC3 and docetaxel-resistant PC3-DR are shown here, representing all mentioned cell lines. For better visualization in the following experiments, only doxycycline-treated shCtrl, shp300-1, and shp300-2 replicates are shown. Confirmation of (A) uniform expression of shRNA-constructs by fluorescence microscopy (BF = bright field, GFP = green fluorescent protein) and magnification 40× and (B) p300 knockdown by Western blot analysis after activation of shp300-sequences with 100 ng/mL doxycycline for 72 h. Values indicated are mean + s.e.m. (one-way ANOVA, *n* = 3). (C) Decreased p300 activity was confirmed by analysis of histone h3 acetylation on lysine18 in PC3 and PC3-DR cells. One representative Western blot out of three independent experiments is shown. (D) Cumulative population doubling levels (PDL, *n* = 1) of PC3 and PC3-DR cells over time with downregulated p300 were calculated by cell number measurement upon each passage (linear fit regression and test for significance of slopes and intercepts).

To study a possible mechanistic role of p300 in docetaxel resistance, PC3, CWR22RV1, and their docetaxel-resistant counterparts were stably transduced with doxycycline-inducible short hairpin (sh)p300 vectors. We selected these cell lines to include both AR-negative (PC3 and PC3-DR) as well as AR-positive (CWR22RV1 and CWR22RV1-DR) sublines. Doxycycline treatment activated the inducible system as indicated by homogeneous expression of vector-integrated GFP (Fig. 3A), which led to decreased p300 protein expression (Fig. 3B) and activity by decreased acetylation of histone h3 on lysine 18 (Fig. 3C). Of note, p300 knockdown had no biologically consistent effect on the proliferation rate of docetaxel-sensitive or docetaxel–resistant cells over a period of 20 days (Fig. 3D). The significantly reduced proliferation rate of PC3-DR shp300-2 was not reproducible with the shp300-1 construct and is, thus, likely an artifact or off-target effect. To exclude the possibility that CBP is upregulated by p300 inhibition, thus compensating for the effects of p300 downregulation, we analyzed CBP expression upon p300 knockdown. As expected, specific downregulation of p300 had no impact on CBP expression (Supplementary Fig. 6).

**Figure 4:**
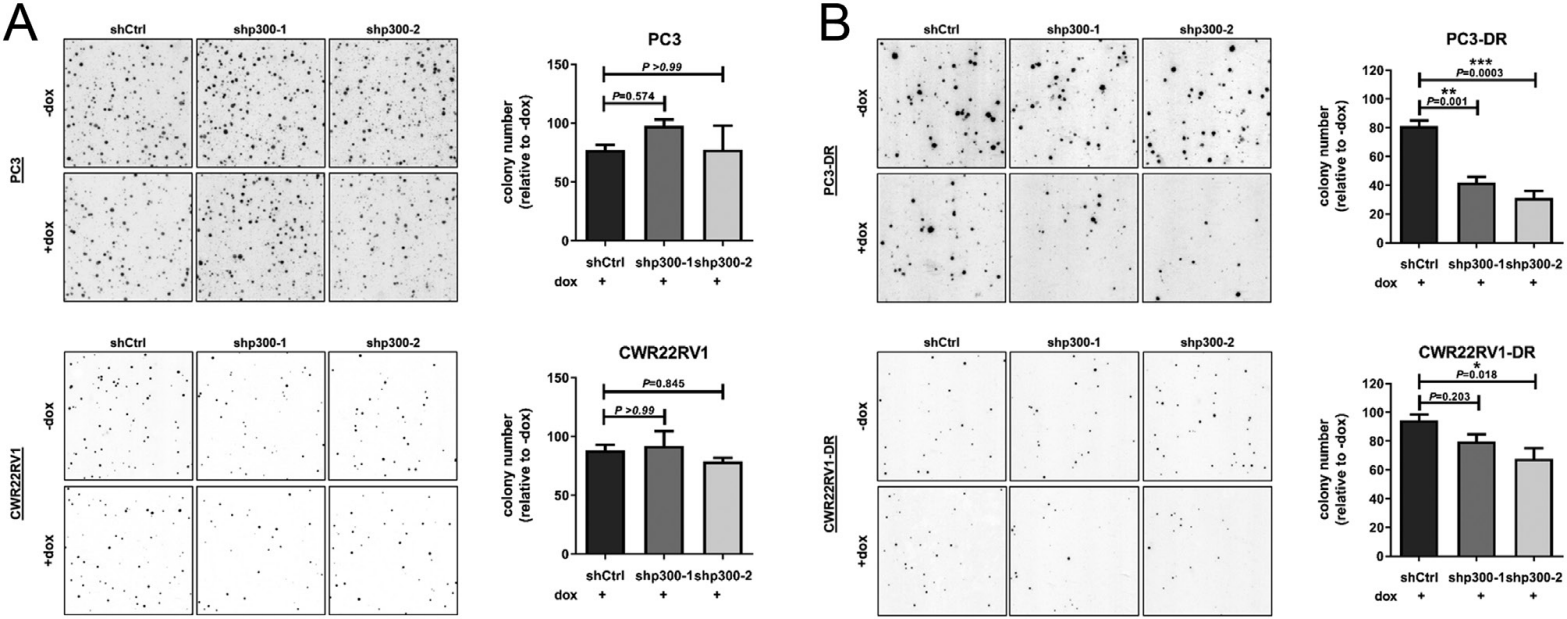
p300 inhibition decreases colony formation ability of docetaxel-resistant cells. Representative images and quantification of high-resolution colony formation assays of docetaxel-sensitive PC3 and CWR22RV1 (A) and docetaxel-resistant PC3-DR and CWR22RV1-DR (B). Colony numbers were analyzed by the software CATCH-colonies. Data represent mean + s.e.m. (one-way ANOVA, *n* = 3).

An important characteristic of aggressive tumor cells is the ability to form colonies, thereby assessing a single cell’s ability to undergo unlimited division. In docetaxel-sensitive PC3 and CWR22RV1, knockdown of p300 had no significant effect on the clonogenic potential (Fig. 4A). However, interestingly, p300 inhibition in docetaxel-resistant PC3-DR and CWR22RV1-DR significantly reduced colony formation efficiency (reduction by 40–50% in PC3-DR and 20–30% in CWR22RV1-DR; Fig. 4B).

### Cell migration and invasion are impaired upon p300 downregulation in docetaxel-resistant cells

**Figure 5:**
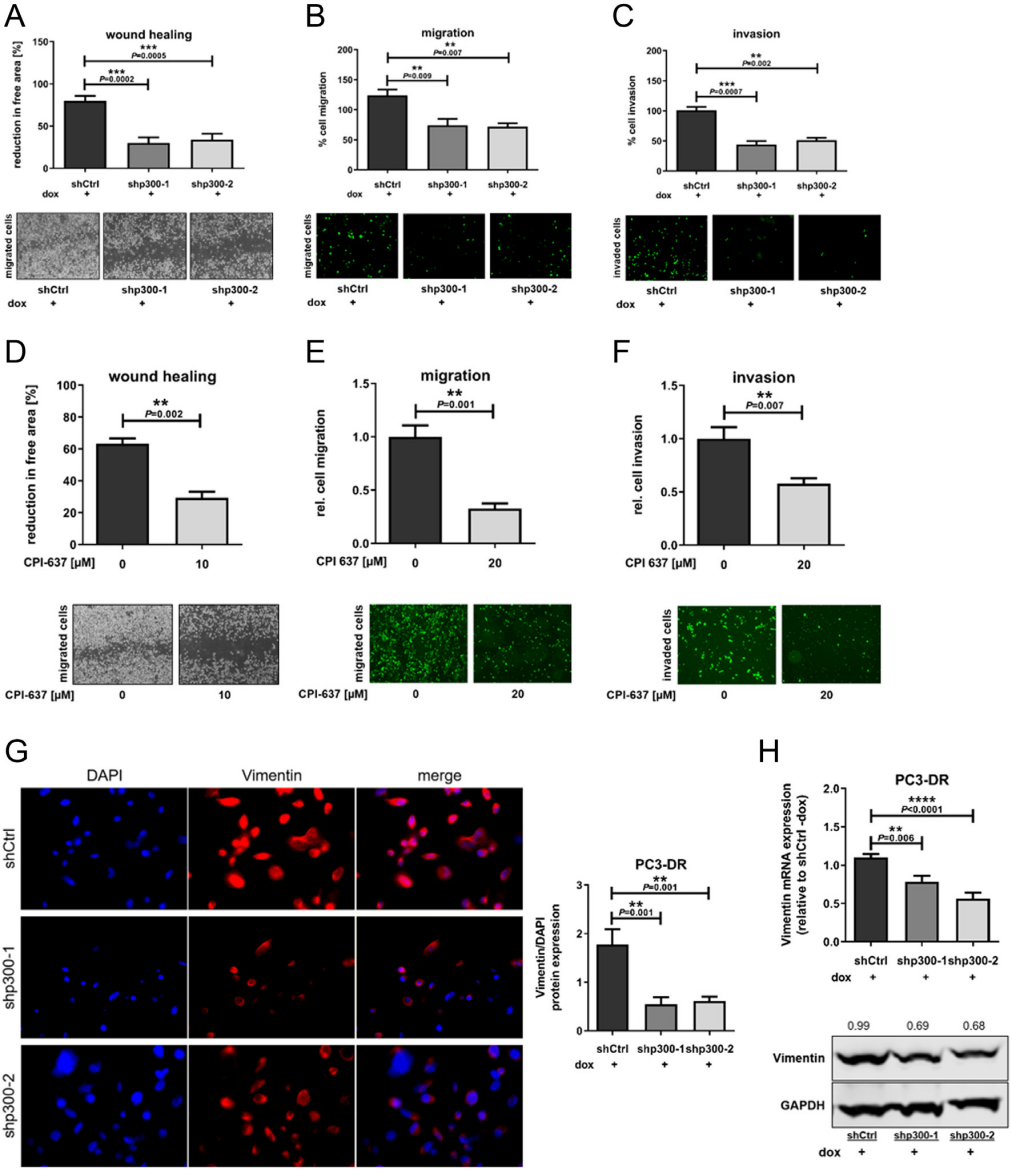
p300 downregulation decreases cell migration and invasion. (A) Wound scratch assays were performed on confluent layers of PC3-DR shCtrl, shp300-1, and shp300-2 treated with 100 ng/mL doxycycline. Images were taken 48 h after scratch and analyzed by ImageJ (*n* = 6). (B) PC3-DR were seeded in Boyden chambers and shp300 sequences were activated with 100 ng/mL doxycycline for 72 h. Cell migration was measured after staining with calcein and visualized by fluorescence microscopy (*n* = 4). (C) Invasion assays were conducted as in B, except that Boyden chambers were pre-coated with Matrigel (*n* = 3). Values indicated in A–C denote mean + s.e.m. (one-way ANOVA). (D) Wound scratch assays on PC3-DR treated with 10 µM CPI-637. Data represent mean + s.e.m. (*t-*Test, *n* = 3). (E) Migration (*n* = 4) and (F) invasion assays (*n* = 5) of PC3-DR treated with 20 µM CPI-637. Values indicated are mean + s.e.m. (*t-*test). (G) Immunofluorescence staining for vimentin (red) and quantification relative to counterstaining of nuclei (blue). Original magnification 630×. (H) mRNA (qPCR) and protein expression (Western blot) of vimentin. Data in G-H represent mean + s.e.m. (one-way ANOVA, *n* = 5).

It has previously been shown that p300 is involved in migration and invasion in PCa cells (Santer *et al.* 2011). Therefore, we wanted to test if p300 inhibition might also impair cell migration and invasion in docetaxel-resistant cells. We employed PC3-DR cells for these experiments since the migration ability of CWR22RV1-DR cells is low. We first performed wound scratch assays that revealed a 2.5-fold decrease in the wound healing rate upon p300 downregulation (Fig. 5A). Consistently, significantly decreased cell migration by ~50% was observed in Boyden chambers following p300 downregulation (Fig. 5B). Moreover, invasion assays, which were conducted on matrigel-coated membranes, also showed a significantly reduced invasion ability (reduction by 50%) upon p300 downregulation (Fig. 5C). These findings were confirmed by the use of the dual inhibitor of p300 and CBP CPI-637. Viability assays were performed to calculate the IC50 for PC3-DR cells (Supplementary Fig. 7). It was determined that IC50 for CPI-637 is 17.52 µM. PC3-DR cells treated with CPI-637 for 72 h revealed a significantly decreased migration and invasion ability (reduction by 50–60%, Fig. 5D, E and F). Vimentin is a cytoskeleton component that plays an important role in migration and was therefore analyzed in p300-silenced PC3-DR cells by immunofluorescence, where we observed a significantly decreased vimentin protein expression (by 60–70%, Fig. 5G). Reduced vimentin levels were additionally confirmed by qRT-PCR and Western blot analysis (Fig. 5H).

## Discussion

Although chemotherapy for CRPC patients confers a clinical benefit for patients, there is no curative treatment available for late stages of PCa. Development of chemotherapy resistance occurs rapidly, and it remains largely unclear which factors are differentially expressed during chemotherapy and might contribute to docetaxel insensitivity. Thus, it is important to understand the molecular mechanisms and to find new therapeutic targets to overcome therapy failure.

As a coactivator of the AR, p300 is involved in many biological processes such as differentiation, proliferation, and cell cycle regulation (Iyer *et al.* 2004) and has already been associated with tumor progression and poor prognosis (Debes *et al.* 2003). Furthermore, p300 expression is increased upon androgen deprivation (Heemers *et al.* 2007) and plays an essential role in ligand-independent transactivation of the AR in androgen-independent PCa cells (Debes *et al.* 2002).

The main finding of this study is that p300 is upregulated upon docetaxel treatment in primary PCa and mCRPC tissue samples as well as in docetaxel-sensitive and docetaxel-resistant PCa cells. IHC staining was performed in samples of patients who received neoadjuvant docetaxel therapy at the Department of Urology of the Medical University of Innsbruck. Of course, these samples do not reflect the same clinical stage as docetaxel-resistant cells. However, patients who receive chemotherapy for PCa in the Authors’ institution are not subjected to removal of tumor tissue. Therefore, the samples from individuals who received neoadjuvant docetaxel therapy were selected to be able to analyze p300 expression in patient material. Furthermore, metastatic tissue samples obtained by rapid autopsy from a publicly available transcriptome dataset including mCRPC patients that received docetaxel in their treatment course were analyzed. Increased 300 expression was observed *in vitro* in docetaxel-resistant AR-positive and AR-negative cells (PC3-DR and CWR22RV1-DR) compared to their respective docetaxel-sensitive counterparts. This finding indicates that p300 is upregulated by docetaxel regardless of AR expression and is consistent with previous publications documenting multiple functions of p300 in PCa also independently of the AR (Debes *et al.* 2003, Santer *et al.* 2011). This is also in concordance with the finding that AR expression and activity are not changed upon docetaxel treatment; although, in previous studies it has been shown that docetaxel impairs transcriptional activity of the AR (Zhu *et al.* 2010). Other groups reported an inhibitory effect of docetaxel on AR activity by interfering with AR intracellular trafficking (Martin *et al.* 2015) and nuclear translocation (Thadani-Mulero *et al.* 2014).

Contrary to p300, the closely related coactivator CBP was not upregulated upon docetaxel treatment. Thus, we conclude that docetaxel-induced upregulation is specific for p300. Hatano and colleagues reported a role of c-Myc, which is a known downstream target of p300 in docetaxel resistance (Hatano *et al.* 2013). Concordant with their study, we observed an increased expression of c-Myc in docetaxel-treated patients; however, the upregulation was not statistically significant. Nevertheless, Myc activity was significantly increased in patients who received docetaxel. Ogiwara and colleagues also reported that p300 ablation caused downregulation of Myc expression in CBP-deficient cells and thereby suppressed cancer cell growth (Ogiwara *et al.* 2016). We assume that p300 upregulation by docetaxel is not mediated by c-Myc, but c-Myc is affected in consequence as a target gene of p300.

We found that p300 mRNA expression was not significantly changed upon docetaxel treatment, which indicates that p300 protein increase is not due to increased transcriptional activity. p300 protein expression also does not increase because of elevated translation, since p300 protein expression in docetaxel-resistant cells was unchanged upon translation inhibition, which suggests that p300 is protected from proteasomal degradation. In a previous study, it was found that androgens downregulate p300 protein but not mRNA expression (Heemers *et al.* 2007). Those results suggest similarities in regulation of p300 by docetaxel and androgens.

It has been previously shown that the competitive histone acetyltransferase p300/CBP inhibitor C646 reduced colony formation in AML cell lines and primary blasts (Gao *et al.* 2013). While this highlights the capacities of HAT inhibitors, there are issues with the potency and selectivity of these early inhibitors (Lasko *et al.* 2017). Meanwhile, more effective bromodomain and extra-terminal (BET) inhibitors that prevent protein-protein interactions between BET proteins and acetylated histones have been developed. The BET inhibitors INCB054329 and INCB057643 have been shown to be effective as single agents in PCa. Likely, novel BET inhibitors will be combined with existing therapies, in particular, for therapy-resistant PCa (Vazquez *et al.* 2019). In this study, we initially employed a shRNA approach to down-regulate p300 and exclude non-specific effects. We confirmed effects of shRNA-mediated p300 downregulation on migration ability of docetaxel-resistant cells with the bromodomain inhibitor CPI-637 that is specific for p300 and CBP, where we observed similar results as with genetic p300 downregulation.

In contrast to docetaxel-sensitive cells, p300 inhibition in docetaxel-resistant PC3-DR and CWR22RV1-DR significantly reduced colony formation efficiency. Downregulation of p300 had no specific effects on proliferation of docetaxel-resistant cells, indicating that p300 inhibition indeed impaired the colony-initiating capacity and that the reduced colony number is not just a secondary effect. It has already been described that docetaxel-resistant cells show a stem-cell-like phenotype (Puhr *et al.* 2012, Marin-Aguilera *et al.* 2014), suggesting that p300 inhibition is effective in conditions in which pathways connected to colony formation and tumor-initiation play a central role. A possible explanation as to why p300 inhibition shows no effects on colony formation of docetaxel-sensitive cells could be the formation of p300-complexes. It has been described that the cysteine protease USP24 stabilizes p300 and thereby increases acetylation of histone h3 (Wang *et al.* 2018). The CtBP1-p300-FOXO 3a complex was found to repress apoptotic regulators Bax and Bim in osteosarcoma cells (Li *et al.* 2019). Cell proliferation is regulated by p300 in complex with the transcriptional repressor YY1 and HDAC2 (Tang *et al.* 2019). Future studies of therapy resistance in PCa should therefore examine and quantitate the complexes relevant to p300. In contrast to PCa, in some models of breast cancer, p300 is considered a tumor inhibitor (Asaduzzaman *et al.* 2017). However, the histone methyltransferase DOT1L in complex with p300 and c-Myc enhanced cellular stemness (Cho *et al.* 2015). The reasons for obviously contrasting effects of p300 on stemness in breast cancer are not yet known.

p300 inhibition significantly decreased cell migration and invasion in PC3-DR cells and reduced vimentin expression. These findings indicate that p300 is involved in cellular pathways that regulate migration and invasion in PCa and are in concordance with previous studies, where they reported involvement of p300 in migration in nasopharyngeal carcinoma and breast cancer cells (Fermento *et al.* 2014, Liao *et al.* 2017).

Taken together, p300 could be a valid target in docetaxel-resistant PCa as it reduces the metastatic potential of docetaxel-resistant cells by reducing colony formation, migration, and invasion capability. So far, multiple factors that contribute to the development of docetaxel resistance in PCa have been described (Patterson *et al.* 2006, Deng *et al.* 2019, Kapur *et al.* 2019). Therefore, docetaxel resistance in PCa is heterogeneous, thus suggesting that medical intervention may be based on a personalized approach. This issue is particularly relevant because p300 inhibitors enter clinical trials in oncology, thus pointing to appropriate selection of patients who will benefit from specific p300 targeting in combinations with other drugs approved or tested for treatment of PCa.

## Supplementary Material

Supplementary figure 1 Verification of p300 antibody specificity. PC3-DR shp300 cells were treated with 100 ng/mL doxycycline for 72 hours to induce p300 knockdown and p300 expression was analyzed by (A) Western Blot (including CBP to exclude cross-reaction of p300 antibody) and (B) IHC staining of p300, magnification 40x. (C) Nuclear localization (arrows) of p300 staining was confirmed by IHC. Magnification 63x.

Supplementary figure 2 Effects of docetaxel on AR expression and activity. (A) AR mRNA expression was analyzed in samples of docetaxel-treated patients compared to control patients (Mann-Whitney U test; box whisker plot with 5-95 percentile). (B) AR activity was assessed by measuring expression scores of the Hallmark Androgen response (Mann-Whitney U test; box whisker plot with 5-95 percentile).

Supplementary figure 3 Expression of p300 and CBP in docetaxel-resistant prostate cancer cells and upon docetaxel treatment. (A - B) Comparison of p300 and CBP mRNA expression between docetaxel-sensitive and docetaxel-resistant (DR) PC3, DU145 and CWR22RV1. Values were normalized to the average signal intensity and represent mean + SEM (t-test, n=3). (C) CBP protein expression in docetaxel-sensitive and docetaxel-resistant cells was determined by Western Blot and one representative Western Blot out of three independent experiments is shown.

Supplementary figure 4 Kinetics of p300 mRNA and protein expression upon docetaxel treatment. (A) PC3, DU145 and CWR22RV1 were treated with the indicated concentrations of docetaxel for 72 hours and p300 mRNA expression was measured by qPCR. Values represent mean + SEM (one-way ANOVA, n=3). PC3 cells were treated with 1 nM docetaxel and p300 mRNA (B) and protein (C) expression were measured at various time points. Data represent mean + SEM (one-way ANOVA, comparing different time points with control 0h, n=3). p300 mRNA (D) and protein (E) expression in docetaxel-resistant PC3-DR cells that were cultured with or without docetaxel were measured by qPCR or Western Blot, respectively. Data represent mean + SEM (t-test; n=3). (F) PC3, docetaxel-treated PC3 and PC3-DR were treated with cycloheximide and p300 expression was measured at the indicated time points. Values represent mean + SEM (one-way ANOVA, comparison of the end points, n=3).

Supplementary figure 5 Expression of c-Myc in patients treated with docetaxel and in cellular models. (A) c-Myc mRNA expression was analyzed in samples of docetaxel-treated patients (Mann-Whitney U test; box whisker plot with 5-95 percentile). (B) Myc activity was assessed by measuring expression scores of the Hallmark Myc targets signatures. (Mann-Whitney U test; box whisker plot with 5-95 percentile). (C) c-Myc protein expression of docetaxel-resistant PC3-DR, DU145-DR and CWR22RV1-DR compared to docetaxel-sensitive counterparts. Data represent mean + SEM. (t-test, n=3). (D) PC3 (n=3), DU145 (n=4) and CWR22RV1 (n=4) were treated with the indicated concentrations of docetaxel for 72 hours and c-Myc protein expression analyzed by Western Blot. Values represent mean + SEM (one-way ANOVA).

Supplementary figure 6 Effect of p300 down-regulation on CBP expression. CBP protein expression after p300 downregulation in PC3 was analyzed by Western Blot and one representative Western Blot out of three independent experiments is shown.

Supplementary figure 7 IC50 curve for PC3-DR cells after treatment with CPI-637. PC3-DR cells were treated with different concentrations of CPI-637 and normalized to treatment with equal amounts of the solvent DMSO. Viability was measured by RealTime-Glo™ MT Cell Viability Assay. Values represent mean + SEM (n=5).

## Declaration of interest

The authors declare that there is no conflict of interest that could be perceived as prejudicing the impartiality of the research reported.

## Funding

This work was supported by the Austrian Science Fund FWF grant W1101-B12 to Z Culig and P31122 to N Sampson.

## Author contribution statement

M Gruber and L Ferrone performed experiments. M Gruber analyzed data and wrote the first version of the manuscript. M Puhr organized and supervised immunohistochemical analyses and supervised *in vitro* experiments with chemoresistance models. T Furlan performed experiments with the bromodomain inhibitor. F R Santer, T Furlan, I Eder, and N Sampson provided experimental suggestions and analyzed data. M Puhr and G Schäfer established TMA. F Handle established the experimental system, performed the bioinformatic analysis of the transcriptome dataset, and supervised *in vitro* experiments. Z Culig was responsible for conception and design of the study, data analysis, and supervision. All authors contributed to manuscript writing.
