# Supplementary figures and images for: p300 is upregulated by docetaxel and is a target in chemoresistant prostate cancer
Source: Endocr Relat Cancer. 2020 Jan 17;27(3):187–98. doi: 10.1530/ERC-19-0488 (PMC7040497; doi:10.1530/ERC-19-0488)

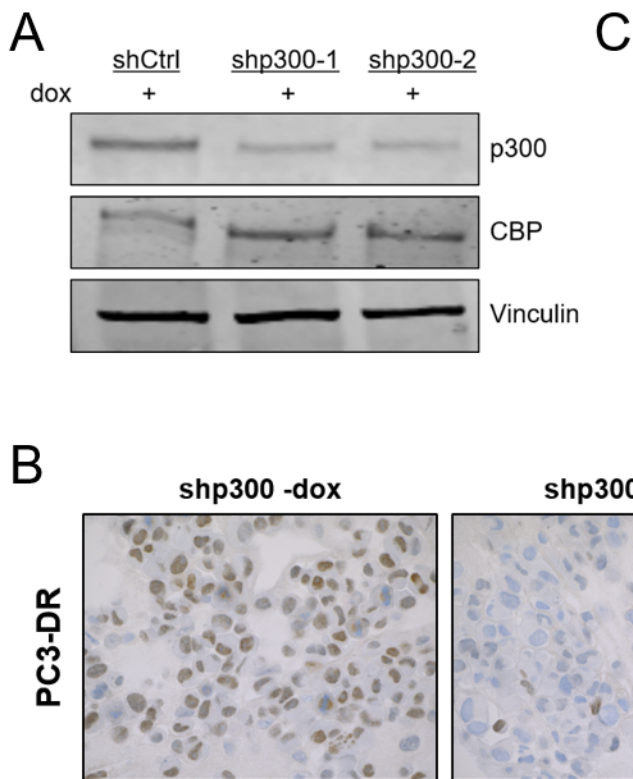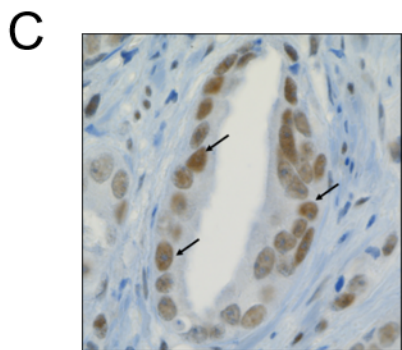

Supplementary figure 1

Supplement: Supplementary figure 1 Verification of p300 antibody specificity. PC3-DR shp300 cells were treated with 100 ng/mL doxycycline for 72 hours to induce p300 knockdown and p300 expression was analyzed by (A) Western Blot (including CBP to exclude cross-reaction of p300 antibody) and (B) IHC staining of  [file supplementary_figure_1.pdf]

## A AR Expression

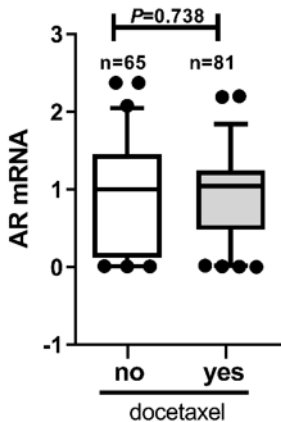

## B Androgen response

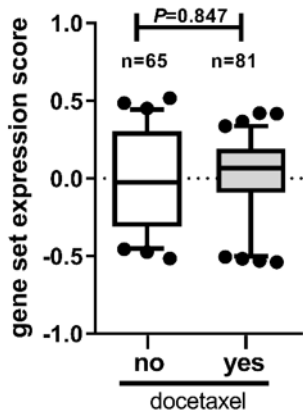

Supplementary figure 2

Supplement: Supplementary figure 2 Effects of docetaxel on AR expression and activity. (A) AR mRNA expression was analyzed in samples of docetaxel-treated patients compared to control patients (Mann-Whitney U test; box whisker plot with 5-95 percentile). (B) AR activity was assessed by measuring expression scor [file supplementary_figure_2.pdf]

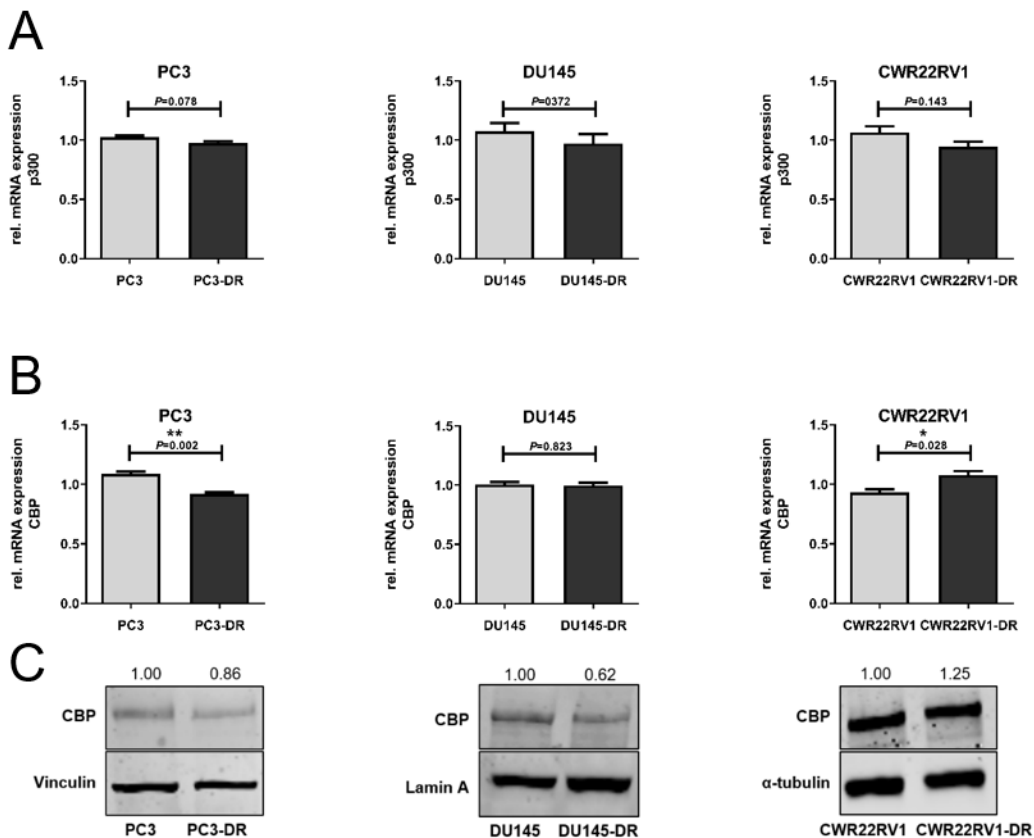

Supplementary figure 3

Supplement: Supplementary figure 3 Expression of p300 and CBP in docetaxel-resistant prostate cancer cells and upon docetaxel treatment. (A - B) Comparison of p300 and CBP mRNA expression between docetaxel-sensitive and docetaxel-resistant (DR) PC3, DU145 and CWR22RV1. Values were normalized to the average sign [file supplementary_figure_3.pdf]

**A**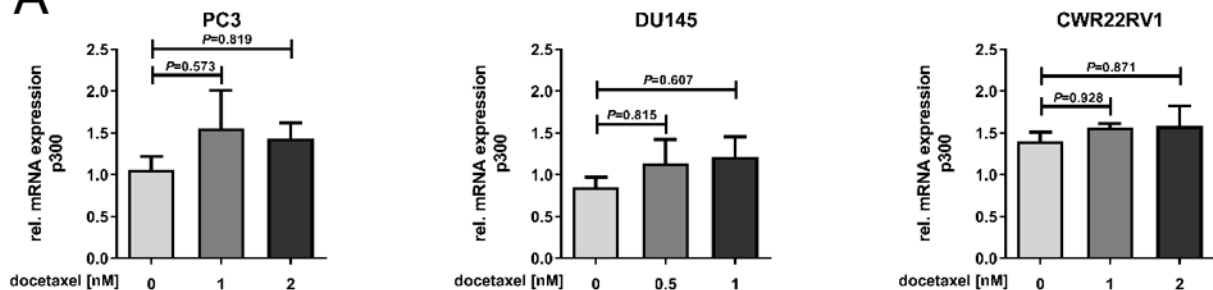**B**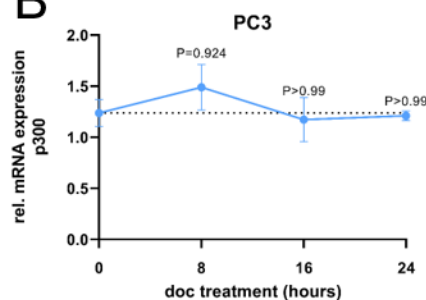**C**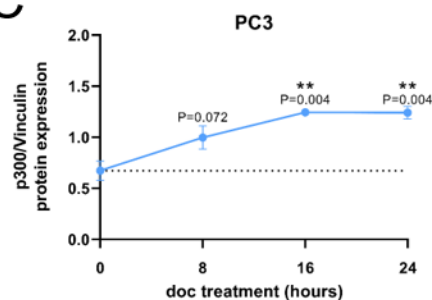**D**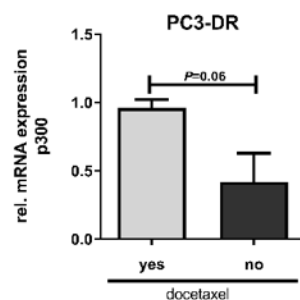**F**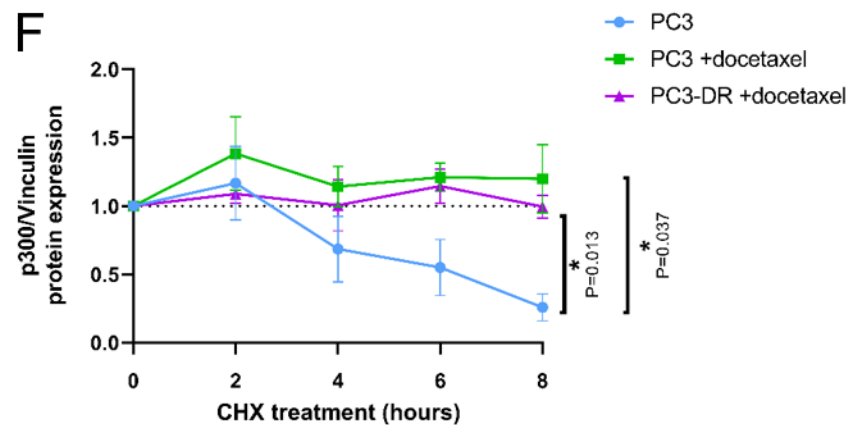**E**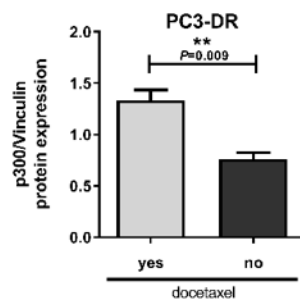

Supplementary figure 4

Supplement: Supplementary figure 4 Kinetics of p300 mRNA and protein expression upon docetaxel treatment. (A) PC3, DU145 and CWR22RV1 were treated with the indicated concentrations of docetaxel for 72 hours and p300 mRNA expression was measured by qPCR. Values represent mean + SEM (one-way ANOVA, n=3). PC3 cell [file supplementary_figure_4.pdf]

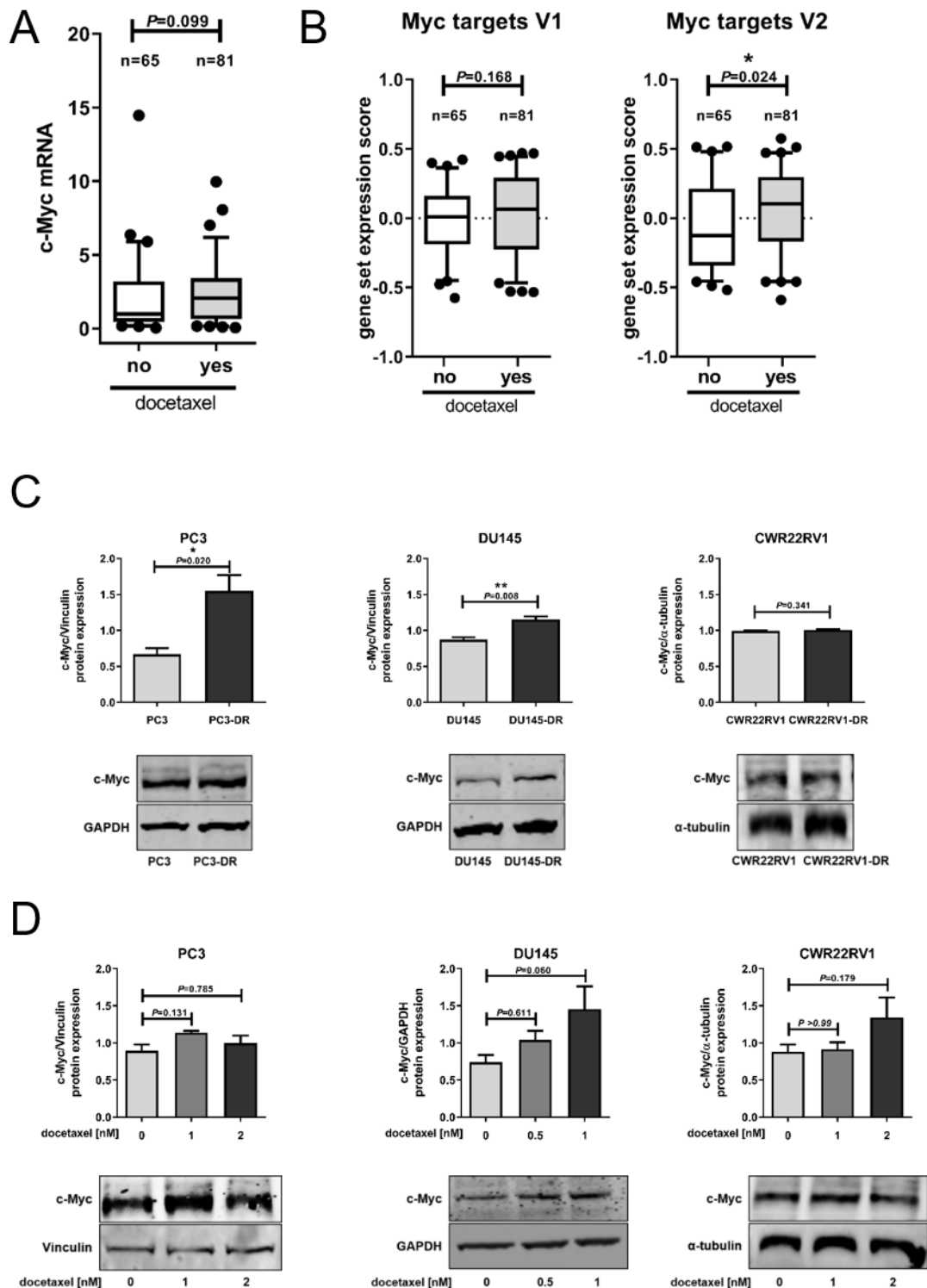

Supplementary figure 5

Supplement: Supplementary figure 5 Expression of c-Myc in patients treated with docetaxel and in cellular models. (A) c-Myc mRNA expression was analyzed in samples of docetaxel-treated patients (Mann-Whitney U test; box whisker plot with 5-95 percentile). (B) Myc activity was assessed by measuring expression sc [file supplementary_figure_5.pdf]

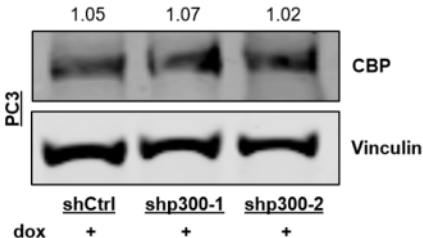

Supplementary figure 6

Supplement: Supplementary figure 6 Effect of p300 down-regulation on CBP expression. CBP protein expression after p300 downregulation in PC3 was analyzed by Western Blot and one representative Western Blot out of three independent experiments is shown. [file supplementary_figure_6.pdf]

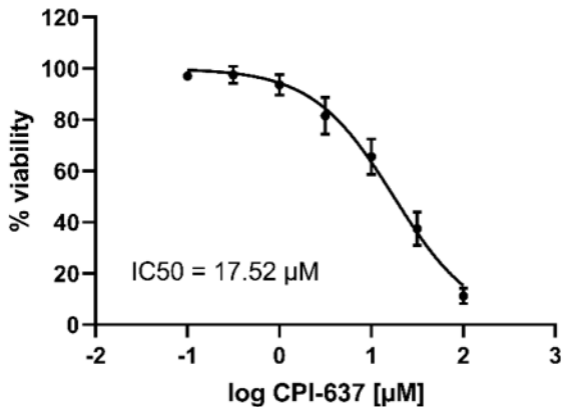

Supplementary figure 7

Supplement: Supplementary figure 7 IC50 curve for PC3-DR cells after treatment with CPI-637. PC3-DR cells were treated with different concentrations of CPI-637 and normalized to treatment with equal amounts of the solvent DMSO. Viability was measured by RealTime-Glo™ MT Cell Viability Assay. Values represent me [file supplementary_figure_7.pdf]
